# Bis(but-2-enoato-κ*O*)tri­phenyl­bis­muth(V)

**DOI:** 10.1107/S1600536813013317

**Published:** 2013-05-18

**Authors:** Pavel V. Andreev, Nikolay V. Somov, Olga S. Kalistratova, Alexey V. Gushchin, Evgeny V. Chuprunov

**Affiliations:** a Department of Physics, N. I. Lobachevsky State University of Nizhni Novgorod, 603950, pr. Gagarina, 23-3 Nizhni Novgorod, Russia; b Department of Chemistry, N. I. Lobachevsky State University of Nizhni Novgorod, 603950, pr. Gagarina, 23-2 Nizhni Novgorod, Russia

## Abstract

In the title mol­ecule, [Bi(C_6_H_5_)_3_(C_4_H_5_O_2_)_2_], the Bi^V^ atom is in a distorted trigonal–bipyramidal environment with carboxyl­ate O atoms in axial positions and phenyl C atoms in the equatorial plane. The Bi—O bond lengths are 2.283 (3) and 2.309 (2) Å, but as a result of additional long Bi⋯O inter­actions [2.787 (3) and 2.734 (3) Å], one of the C—Bi—C angles is 148.62 (13)°. In the crystal, weak C—H⋯O hydrogen bonds connect pairs of mol­ecules into inversion dimers. These dimers are further connected by weak C—H⋯π inter­actions into chains along [100] .

## Related literature

For the isotypic (C_6_H_5_)_3_Sb(C_4_H_5_O_2_)_2_ structure, see: Gushchin *et al.* (2013 ▶). For closely related structures, see: Andreev *et al.* (2013 ▶); Belsky (1996 ▶). For the chemistry of triphenyanti­mony di­acyl­ates, see: Gushchin *et al.* (2011 ▶), for their thermodynamic properties, see: Letyanina *et al.* (2012 ▶); Markin *et al.* (2011 ▶) and for their applications, see: Dodonov & Gushchin (2004 ▶). For van der Waals radii, see: Batsanov (2001 ▶).
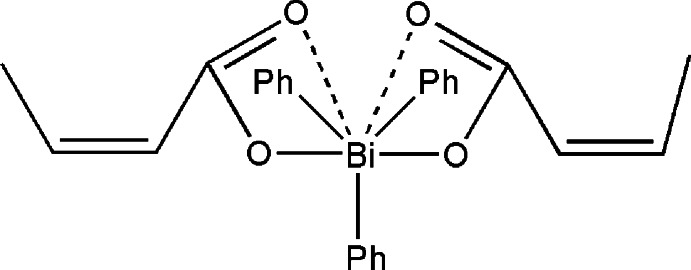


## Experimental

### 

#### Crystal data

[Bi(C_6_H_5_)_3_(C_4_H_5_O_2_)_2_]
*M* *_r_* = 610.44
Triclinic, 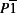

*a* = 10.4710 (3) Å
*b* = 10.4957 (3) Å
*c* = 11.9774 (3) Å
α = 84.941 (2)°
β = 83.633 (2)°
γ = 69.084 (3)°
*V* = 1220.32 (6) Å^3^
*Z* = 2
Mo *K*α radiation
μ = 7.25 mm^−1^
*T* = 293 K
0.09 mm (radius)

#### Data collection

Agilent Xcalibur (Sapphire3, Gemini) diffractometer
Absorption correction: multi-scan (*CrysAlis PRO*; Agilent, 2011 ▶) *T*_min_ = 0.810, *T*_max_ = 1
17262 measured reflections
4946 independent reflections
4620 reflections with *I* > 2σ(*I*)
*R*_int_ = 0.024

#### Refinement

*R*[*F* ^2^ > 2σ(*F* ^2^)] = 0.020
*wR*(*F* ^2^) = 0.050
*S* = 1.11
4946 reflections
280 parameters
2 restraints
H-atom parameters constrained
Δρ_max_ = 0.65 e Å^−3^
Δρ_min_ = −0.84 e Å^−3^

### 

Data collection: *CrysAlis PRO* (Agilent, 2011 ▶); cell refinement: *CrysAlis PRO*; data reduction: *CrysAlis RED* (Agilent, 2011 ▶); program(s) used to solve structure: *SHELXS97* (Sheldrick, 2008 ▶); program(s) used to refine structure: *SHELXL97* (Sheldrick, 2008 ▶); molecular graphics: *Mercury* (Macrae *et al.*, 2006 ▶); software used to prepare material for publication: *publCIF* (Westrip, 2010 ▶).

## Supplementary Material

Crystal structure: contains datablock(s) I, global. DOI: 10.1107/S1600536813013317/lh5612sup1.cif

Structure factors: contains datablock(s) I. DOI: 10.1107/S1600536813013317/lh5612Isup2.hkl

Additional supplementary materials:  crystallographic information; 3D view; checkCIF report

## Figures and Tables

**Table 1:** Hydrogen-bond geometry (Å, °)

*D*—H⋯*A*	*D*—H	H⋯*A*	*D*⋯*A*	*D*—H⋯*A*
C3—H3⋯O2*A* ^i^	0.93	2.53	3.450 (6)	171
C4*B*—H4*B*1⋯C1*A* ^ii^	0.96	2.74	3.683 (6)	167
C4*B*—H4*B*1⋯C2*A* ^ii^	0.96	2.85	3.613 (7)	137

Symmetry codes: (i) 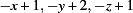; (ii) 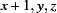.

**Fig. 1.**
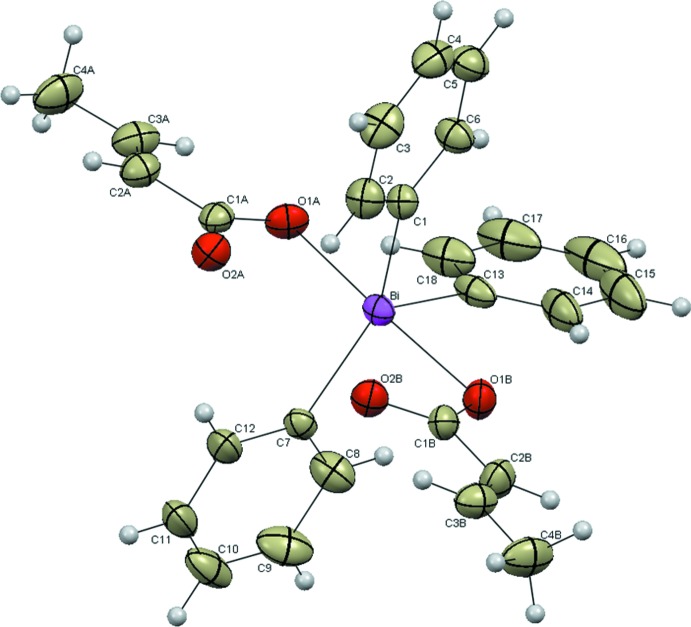
The molecular structure of the title compound. Displacement ellipsoids are drawn at the 30% probability level. H atoms are presented as small spheres of arbitrary radius.

**Fig. 2.**
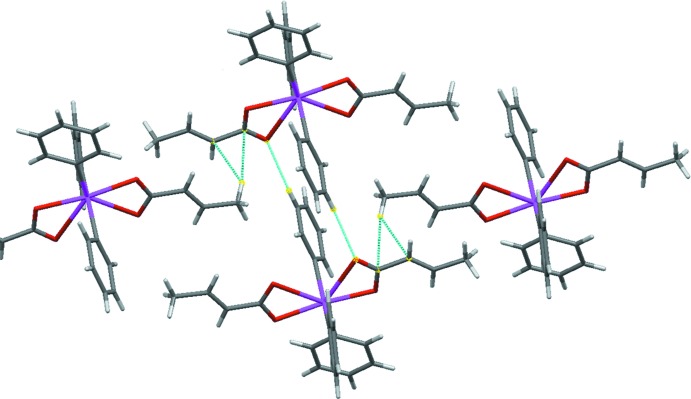
Part of the crystal structure with weak C—H···O and C—H···π interactions shown as dashed lines connecting moleclues along [100].

## supplementary crystallographic information

### Comment

Bis(but-2-enoate) triphenylbismuth C_26_H_25_O_4_Bi belongs to the family of triphenylbismuth diacylates. Compounds of triphenylbismuth and triphenylantimony diacylates contain two double bonds C═C in the molecule, due to which they can be used for polymerization filling of polystyrene and polymethylmethacrylate. The title compound is a very promising monomer for developing metal-containing organic scintillators which have recently attracted much attention in high-energy physics. It was found that the participation of both acrylate groups in polymerization leads to cross-linking, considerably decreasing the thermooxidative destruction of the resulting polymer (Dodonov *et al.*, 2004). Organic glasses based on triphenylantimony diacrylate and methylmethacrylate having increased fungal resistance are now available (Dodonov *et al.*, 2004). The thermodynamic properties of triphenylantimony diacylates have been studied (Letyanina *et al.*, 2012; Markin *et al.*, 2011). The crystal structure and chemistry of a similar orgaonmetallic compound of antimony has been reported by Gushchin *et al.* (2013).

In the molecule of title compound the O—Bi—O angle is 172.64 (9)° and the C_phenyl_—Bi—C_phenyl_ angles are 148.62 (13)°, 106.20 (13)°, 150.10 (13)°. Such a deviation for the latter from the ideal 120° is typical for triphenylantomony diacylates because of additional long Bi···O interactions. A similar geometries were observed in triphenylantomony diacylates (Gushchin *et al.*, 2011; Gushchin *et al.*, 2013; Andreev *et al.*, 2013). The Bi–O2A and Bi–O2B distances are 2.787 (3) Å and 2.734 (3) Å, respectively and are significantly shorter than the sum of the van der Waals radii of these atoms (3.85 Å) (Batsanov, 2001). A similar interaction was observed in triphenylantimony dimetacrylate (Gushchin *et al.*, 2011), bis[(*E*)-3-(4-methoxyphenyl)prop-2-enoato]triphenylantimony(V) (Andreev *et al.*, 2013), triphenylantimony dicrotonate (Gushchin *et al.*, 2013) and triphenylantimony-bis(cinnamate) (Belsky, 1996). In the crystal, weak C—H···O hydrogen bonds connect pairs of molecules into inversion dimers. These dimers are further connected by weak C—H···π interactions into chains along [100] (see Fig.2).

### Experimental

The synthesis was carried out on the oxidation addition reaction of triphenylbismuth, crotonic acid and *tert*-butyl hydroperoxide. To a solution of 0.56 ml of 92.6% aqueous *tert*-butyl hydroperoxide and 0.86 g of crotonic acid in 20 ml of diethyl ether was added a solution of 2.2 g of triphenylbismuth. The mixture was kept for 24 h at room temperature. The yellow crystals formed were filtered off and dried to obtain 1.91 g (73%) of triphenylbismuth bis(but-2-enoate). The product was recrystallized twice from chloroform-hexane mixture (1:4), m.p. 426 K. A crystal for X-ray diffraction analysis was obtained from benzene solution.

### Refinement

H atoms were positioned geometrically (C—H=0.95–1.00 Å) and refined using a riding model with the *U*_iso_(H)=1.2U_eq_(C) (1.5U_eq_(C) for methyl groups). In the refinment the anisotropic displacment parameters of atoms pairs C9/C10 and C16/C17 were restrained using the DELU instruction in SHELXL (Sheldrick, 2008).

### Crystal data

**Table:**

[Bi(C_6_H_5_)_3_(C_4_H_5_O_2_)_2_]	*Z* = 2
*M**_r_* = 610.44	*F*(000) = 592
Triclinic, *P*1	*D*_x_ = 1.661 Mg m^−^^3^
Hall symbol: -P 1	Mo *K*α radiation, λ = 0.71073 Å
*a* = 10.4710 (3) Å	Cell parameters from 9953 reflections
*b* = 10.4957 (3) Å	θ = 3.4–32.8°
*c* = 11.9774 (3) Å	µ = 7.25 mm^−^^1^
α = 84.941 (2)°	*T* = 293 K
β = 83.633 (2)°	Sphere, colourless
γ = 69.084 (3)°	0.09 mm (radius)
*V* = 1220.32 (6) Å^3^	

### Data collection

**Table:**

Agilent Xcalibur (Sapphire3, Gemini) diffractometer	4946 independent reflections
Graphite monochromator	4620 reflections with *I* > 2σ(*I*)
Detector resolution: 16.0302 pixels mm^-1^	*R*_int_ = 0.024
ω scans	θ_max_ = 26.4°, θ_min_ = 3.4°
Absorption correction: multi-scan (*CrysAlis PRO*; Agilent, 2011)	*h* = −13→13
*T*_min_ = 0.810, *T*_max_ = 1	*k* = −13→13
17262 measured reflections	*l* = −14→14

### Refinement

**Table:**

Refinement on *F*^2^	Primary atom site location: structure-invariant direct methods
Least-squares matrix: full	Secondary atom site location: difference Fourier map
*R*[*F*^2^ > 2σ(*F*^2^)] = 0.020	Hydrogen site location: inferred from neighbouring sites
*wR*(*F*^2^) = 0.050	H-atom parameters constrained
*S* = 1.11	*w* = 1/[σ^2^(*F*_o_^2^) + (0.0212*P*)^2^ + 0.9058*P*] where *P* = (*F*_o_^2^ + 2*F*_c_^2^)/3
4946 reflections	(Δ/σ)_max_ = 0.001
280 parameters	Δρ_max_ = 0.65 e Å^−^^3^
2 restraints	Δρ_min_ = −0.84 e Å^−^^3^

### Special details

**Table:**

Geometry. All s.u.'s (except the s.u. in the dihedral angle between two l.s. planes) are estimated using the full covariance matrix. The cell e.s.d.'s are taken into account individually in the estimation of e.s.d.'s in distances, angles and torsion angles; correlations between e.s.d.'s in cell parameters are only used when they are defined by crystal symmetry. An approximate (isotropic) treatment of cell e.s.d.'s is used for estimating e.s.d.'s involving l.s. planes.
Refinement. Refinement of *F*^2^ against ALL reflections. The weighted *R*-factor *wR* and goodness of fit *S* are based on *F*^2^, conventional *R*-factors *R* are based on *F*, with *F* set to zero for negative *F*^2^. The threshold expression of *F*^2^ > σ(*F*^2^) is used only for calculating *R*-factors(gt) *etc*. and is not relevant to the choice of reflections for refinement. *R*-factors based on *F*^2^ are statistically about twice as large as those based on *F*, and *R*- factors based on ALL data will be even larger.

### Fractional atomic coordinates and isotropic or equivalent isotropic displacement parameters (Å^2^)

**Table:**

	*x*	*y*	*z*	*U*_iso_*/*U*_eq_	
Bi	0.714269 (12)	0.636573 (11)	0.737494 (10)	0.03886 (5)	
O1A	0.4935 (3)	0.6470 (3)	0.7972 (2)	0.0561 (7)	
O1B	0.9413 (3)	0.5981 (3)	0.6880 (2)	0.0595 (7)	
O2A	0.4728 (3)	0.8624 (3)	0.7607 (2)	0.0574 (7)	
O2B	0.8411 (3)	0.8212 (3)	0.6718 (2)	0.0552 (6)	
C1	0.6556 (3)	0.6872 (3)	0.5635 (3)	0.0416 (7)	
C2	0.6338 (4)	0.8145 (4)	0.5113 (3)	0.0564 (9)	
H2	0.6465	0.8833	0.5476	0.068*	
C1A	0.4227 (4)	0.7768 (4)	0.7950 (3)	0.0479 (8)	
C1B	0.9455 (4)	0.7179 (4)	0.6631 (3)	0.0485 (8)	
C3	0.5922 (5)	0.8372 (5)	0.4026 (4)	0.0679 (12)	
H3	0.5777	0.922	0.3652	0.082*	
C2A	0.2745 (4)	0.8208 (5)	0.8375 (3)	0.0598 (10)	
H2A	0.2218	0.9128	0.8278	0.072*	
C2B	1.0828 (4)	0.7244 (5)	0.6218 (4)	0.0641 (11)	
H2B	1.1585	0.644	0.6247	0.077*	
C4	0.5722 (4)	0.7356 (5)	0.3504 (3)	0.0663 (12)	
H4	0.5431	0.7525	0.2782	0.08*	
C3A	0.2156 (5)	0.7417 (6)	0.8858 (4)	0.0697 (12)	
H3A	0.2683	0.6494	0.8926	0.084*	
C3B	1.1031 (4)	0.8329 (5)	0.5829 (3)	0.0617 (11)	
H3B	1.0262	0.9124	0.5813	0.074*	
C5	0.5947 (5)	0.6103 (5)	0.4032 (4)	0.0650 (11)	
H5	0.5815	0.5421	0.3664	0.078*	
C4A	0.0675 (5)	0.7837 (6)	0.9334 (4)	0.0850 (16)	
H4A1	0.0634	0.753	1.0111	0.127*	
H4A2	0.0192	0.7435	0.8922	0.127*	
H4A3	0.0258	0.8814	0.927	0.127*	
C4B	1.2384 (5)	0.8463 (6)	0.5391 (4)	0.0845 (16)	
H4B3	1.2241	0.9396	0.5143	0.127*	
H4B2	1.2778	0.7886	0.477	0.127*	
H4B1	1.2995	0.8194	0.5979	0.127*	
C6	0.6372 (4)	0.5830 (4)	0.5115 (3)	0.0553 (9)	
H6	0.6529	0.4975	0.5478	0.066*	
C7	0.7323 (3)	0.7074 (4)	0.9006 (3)	0.0424 (7)	
C8	0.7989 (5)	0.6056 (5)	0.9773 (3)	0.0664 (11)	
H8	0.8324	0.5141	0.9602	0.08*	
C9	0.8137 (6)	0.6466 (7)	1.0826 (4)	0.0850 (16)	
H9	0.8588	0.5815	1.1364	0.102*	
C10	0.7615 (6)	0.7831 (7)	1.1063 (4)	0.0817 (16)	
H10	0.7706	0.8091	1.1764	0.098*	
C11	0.6975 (5)	0.8790 (6)	1.0283 (5)	0.0787 (14)	
H11	0.6639	0.9705	1.0452	0.094*	
C12	0.6811 (4)	0.8437 (4)	0.9245 (4)	0.0620 (11)	
H12	0.6365	0.9102	0.8714	0.074*	
C13	0.7740 (5)	0.4106 (4)	0.7598 (3)	0.0580 (11)	
C14	0.8981 (6)	0.3282 (4)	0.7091 (4)	0.0794 (15)	
H14	0.9579	0.367	0.6695	0.095*	
C15	0.9322 (8)	0.1884 (5)	0.7176 (6)	0.105 (2)	
H15	1.0151	0.1318	0.684	0.126*	
C16	0.8411 (10)	0.1335 (6)	0.7771 (6)	0.121 (3)	
H16	0.8641	0.0391	0.7828	0.145*	
C17	0.7176 (8)	0.2141 (6)	0.8281 (6)	0.105 (2)	
H17	0.6576	0.1751	0.8671	0.126*	
C18	0.6848 (6)	0.3539 (5)	0.8202 (4)	0.0782 (15)	
H18	0.6027	0.4101	0.8553	0.094*	

### Atomic displacement parameters (Å^2^)

**Table:**

	*U*^11^	*U*^22^	*U*^33^	*U*^12^	*U*^13^	*U*^23^
Bi	0.04616 (8)	0.02998 (7)	0.04346 (8)	−0.01683 (5)	−0.00534 (5)	−0.00091 (5)
O1A	0.0565 (16)	0.0633 (17)	0.0606 (16)	−0.0385 (14)	−0.0047 (12)	0.0079 (13)
O1B	0.0508 (15)	0.0522 (16)	0.0720 (18)	−0.0142 (12)	0.0046 (13)	−0.0134 (14)
O2A	0.0568 (16)	0.0524 (16)	0.0637 (17)	−0.0219 (13)	−0.0041 (13)	0.0029 (13)
O2B	0.0543 (16)	0.0501 (15)	0.0643 (17)	−0.0235 (13)	−0.0009 (12)	−0.0026 (12)
C1	0.0444 (18)	0.0370 (17)	0.0413 (18)	−0.0136 (14)	−0.0008 (14)	0.0014 (14)
C2	0.064 (2)	0.046 (2)	0.060 (2)	−0.0233 (18)	−0.0027 (19)	0.0064 (18)
C1A	0.0438 (19)	0.059 (2)	0.0399 (18)	−0.0153 (17)	−0.0087 (15)	−0.0013 (16)
C1B	0.0429 (19)	0.056 (2)	0.0445 (19)	−0.0155 (17)	0.0027 (15)	−0.0108 (16)
C3	0.072 (3)	0.067 (3)	0.057 (3)	−0.021 (2)	−0.004 (2)	0.024 (2)
C2A	0.057 (2)	0.067 (3)	0.056 (2)	−0.021 (2)	−0.0122 (18)	0.003 (2)
C2B	0.045 (2)	0.067 (3)	0.078 (3)	−0.0170 (19)	0.005 (2)	−0.012 (2)
C4	0.057 (2)	0.096 (4)	0.043 (2)	−0.025 (2)	−0.0034 (18)	0.007 (2)
C3A	0.064 (3)	0.092 (3)	0.062 (3)	−0.039 (3)	−0.013 (2)	0.010 (2)
C3B	0.057 (2)	0.087 (3)	0.049 (2)	−0.036 (2)	−0.0039 (18)	0.000 (2)
C5	0.068 (3)	0.078 (3)	0.054 (2)	−0.029 (2)	−0.008 (2)	−0.011 (2)
C4A	0.052 (3)	0.129 (5)	0.078 (3)	−0.042 (3)	0.000 (2)	0.008 (3)
C4B	0.066 (3)	0.133 (5)	0.070 (3)	−0.059 (3)	0.000 (2)	0.005 (3)
C6	0.069 (3)	0.048 (2)	0.053 (2)	−0.0231 (19)	−0.0108 (19)	−0.0015 (17)
C7	0.0435 (18)	0.0462 (19)	0.0433 (18)	−0.0229 (15)	0.0007 (14)	−0.0077 (15)
C8	0.094 (3)	0.063 (3)	0.051 (2)	−0.040 (2)	−0.007 (2)	0.003 (2)
C9	0.116 (4)	0.119 (4)	0.043 (2)	−0.070 (4)	−0.014 (2)	0.011 (3)
C10	0.097 (4)	0.124 (4)	0.050 (3)	−0.071 (4)	0.021 (3)	−0.036 (3)
C11	0.068 (3)	0.089 (4)	0.086 (4)	−0.030 (3)	0.005 (3)	−0.049 (3)
C12	0.056 (2)	0.056 (2)	0.078 (3)	−0.0197 (19)	−0.006 (2)	−0.025 (2)
C13	0.092 (3)	0.0321 (18)	0.056 (2)	−0.0221 (19)	−0.034 (2)	0.0034 (16)
C14	0.111 (4)	0.039 (2)	0.081 (3)	−0.010 (2)	−0.031 (3)	−0.011 (2)
C15	0.156 (6)	0.042 (3)	0.106 (4)	−0.007 (3)	−0.048 (4)	−0.014 (3)
C16	0.206 (8)	0.043 (3)	0.126 (5)	−0.036 (4)	−0.104 (5)	0.017 (3)
C17	0.172 (6)	0.055 (3)	0.114 (5)	−0.061 (4)	−0.079 (4)	0.034 (3)
C18	0.117 (4)	0.049 (2)	0.085 (3)	−0.044 (3)	−0.043 (3)	0.022 (2)

### Geometric parameters (Å, º)

**Table:**

Bi—C7	2.201 (3)	C5—H5	0.93
Bi—C1	2.205 (3)	C4A—H4A1	0.96
Bi—C13	2.226 (4)	C4A—H4A2	0.96
Bi—O1B	2.283 (3)	C4A—H4A3	0.96
Bi—O1A	2.309 (2)	C4B—H4B3	0.96
Bi—O2A	2.787 (3)	C4B—H4B2	0.96
Bi—O2B	2.734 (3)	C4B—H4B1	0.96
O1A—C1A	1.297 (5)	C6—H6	0.93
O1B—C1B	1.281 (5)	C7—C12	1.380 (5)
O2A—C1A	1.215 (4)	C7—C8	1.382 (6)
O2B—C1B	1.237 (4)	C8—C9	1.409 (6)
C1—C2	1.376 (5)	C8—H8	0.93
C1—C6	1.385 (5)	C9—C10	1.381 (8)
C2—C3	1.392 (6)	C9—H9	0.93
C2—H2	0.93	C10—C11	1.352 (8)
C1A—C2A	1.495 (5)	C10—H10	0.93
C1B—C2B	1.491 (5)	C11—C12	1.373 (6)
C3—C4	1.369 (7)	C11—H11	0.93
C3—H3	0.93	C12—H12	0.93
C2A—C3A	1.268 (6)	C13—C14	1.386 (7)
C2A—H2A	0.93	C13—C18	1.386 (6)
C2B—C3B	1.271 (6)	C14—C15	1.377 (7)
C2B—H2B	0.93	C14—H14	0.93
C4—C5	1.360 (6)	C15—C16	1.385 (10)
C4—H4	0.93	C15—H15	0.93
C3A—C4A	1.511 (6)	C16—C17	1.377 (11)
C3A—H3A	0.93	C16—H16	0.93
C3B—C4B	1.506 (6)	C17—C18	1.380 (7)
C3B—H3B	0.93	C17—H17	0.93
C5—C6	1.391 (6)	C18—H18	0.93
			
C7—Bi—C1	148.62 (13)	C3A—C4A—H4A1	109.5
C7—Bi—C13	106.20 (13)	C3A—C4A—H4A2	109.5
C1—Bi—C13	105.10 (13)	H4A1—C4A—H4A2	109.5
C7—Bi—O1B	90.15 (11)	C3A—C4A—H4A3	109.5
C1—Bi—O1B	94.14 (11)	H4A1—C4A—H4A3	109.5
C13—Bi—O1B	86.30 (14)	H4A2—C4A—H4A3	109.5
C7—Bi—O1A	89.80 (11)	C3B—C4B—H4B3	109.5
C1—Bi—O1A	89.73 (11)	C3B—C4B—H4B2	109.5
C13—Bi—O1A	86.65 (14)	H4B3—C4B—H4B2	109.5
O1B—Bi—O1A	172.64 (9)	C3B—C4B—H4B1	109.5
C7—Bi—O2B	78.59 (10)	H4B3—C4B—H4B1	109.5
C1—Bi—O2B	80.12 (11)	H4B2—C4B—H4B1	109.5
C13—Bi—O2B	137.31 (14)	C1—C6—C5	117.9 (4)
O1B—Bi—O2B	51.02 (9)	C1—C6—H6	121
O1A—Bi—O2B	136.05 (9)	C5—C6—H6	121
C1A—O1A—Bi	104.0 (2)	C12—C7—C8	122.4 (4)
C1B—O1B—Bi	103.8 (2)	C12—C7—Bi	122.4 (3)
C1B—O2B—Bi	83.6 (2)	C8—C7—Bi	115.1 (3)
C2—C1—C6	122.2 (3)	C7—C8—C9	117.0 (5)
C2—C1—Bi	122.7 (3)	C7—C8—H8	121.5
C6—C1—Bi	115.1 (2)	C9—C8—H8	121.5
C1—C2—C3	118.0 (4)	C10—C9—C8	120.4 (5)
C1—C2—H2	121	C10—C9—H9	119.8
C3—C2—H2	121	C8—C9—H9	119.8
O2A—C1A—O1A	122.4 (3)	C11—C10—C9	120.4 (4)
O2A—C1A—C2A	119.5 (4)	C11—C10—H10	119.8
O1A—C1A—C2A	118.1 (3)	C9—C10—H10	119.8
O2B—C1B—O1B	121.6 (3)	C10—C11—C12	121.2 (5)
O2B—C1B—C2B	122.4 (4)	C10—C11—H11	119.4
O1B—C1B—C2B	115.9 (3)	C12—C11—H11	119.4
C4—C3—C2	120.5 (4)	C11—C12—C7	118.6 (5)
C4—C3—H3	119.7	C11—C12—H12	120.7
C2—C3—H3	119.7	C7—C12—H12	120.7
C3A—C2A—C1A	124.7 (4)	C14—C13—C18	120.7 (4)
C3A—C2A—H2A	117.6	C14—C13—Bi	119.4 (3)
C1A—C2A—H2A	117.6	C18—C13—Bi	119.9 (3)
C3B—C2B—C1B	124.2 (4)	C15—C14—C13	119.6 (6)
C3B—C2B—H2B	117.9	C15—C14—H14	120.2
C1B—C2B—H2B	117.9	C13—C14—H14	120.2
C5—C4—C3	120.6 (4)	C14—C15—C16	119.0 (7)
C5—C4—H4	119.7	C14—C15—H15	120.5
C3—C4—H4	119.7	C16—C15—H15	120.5
C2A—C3A—C4A	126.0 (5)	C17—C16—C15	122.1 (5)
C2A—C3A—H3A	117	C17—C16—H16	118.9
C4A—C3A—H3A	117	C15—C16—H16	118.9
C2B—C3B—C4B	126.8 (5)	C16—C17—C18	118.6 (7)
C2B—C3B—H3B	116.6	C16—C17—H17	120.7
C4B—C3B—H3B	116.6	C18—C17—H17	120.7
C4—C5—C6	120.7 (4)	C17—C18—C13	120.0 (6)
C4—C5—H5	119.6	C17—C18—H18	120
C6—C5—H5	119.6	C13—C18—H18	120

### Hydrogen-bond geometry (Å, º)

**Table:**

*D*—H···*A*	*D*—H	H···*A*	*D*···*A*	*D*—H···*A*
C3—H3···O2*A*^i^	0.93	2.53	3.450 (6)	171
C4*B*—H4*B*1···C1*A*^ii^	0.96	2.74	3.683 (6)	167
C4*B*—H4*B*1···C2*A*^ii^	0.96	2.85	3.613 (7)	137

Symmetry codes: (i) −*x*+1, −*y*+2, −*z*+1; (ii) *x*+1, *y*, *z*.

## References

[bb1] Agilent (2011). *CrysAlis PRO* and *CrysAlis RED* Agilent Technologies, Yarnton, England.

[bb2] Andreev, P. V., Somov, N. V., Kalistratova, O. S., Gushchin, A. V. & Chuprunov, E. V. (2013). *Acta Cryst.* E**69**, m167. 10.1107/S1600536813004674 PMC3588489 23476508

[bb3] Batsanov, S. S. (2001). *Inorg. Mater.* **37**, 871–885.

[bb4] Belsky, V. K. (1996). Private communication (refcode NAGXOI). CCDC, Cambridge, England.

[bb5] Dodonov, V. A. & Gushchin, A. V. (2004). *Vestn. NNovg. Univ.* **82**, 86–94.

[bb6] Gushchin, A. V., Kalistratova, O. S., Verkhovykh, R. A., Somov, N. V., Shashkin, D. V. & Dodonov, V. A. (2013). *Vestn. NNovg. Univ.* **1**, 86–90.

[bb7] Gushchin, A. V., Shashkin, D. V., Prytkova, L. K., Somov, N. V., Baranov, E. V., Shavyrin, A. S. & Rykalin, V. I. (2011). *Russ. J. General Chem.* **81**, 493–496.

[bb8] Letyanina, I. A., Markin, A. V., Smirnova, N. N., Gushchin, A. V. & Shashkin, D. V. (2012). *Russ. J. Phys. Chem.* **A86**, 1189–1195.

[bb9] Macrae, C. F., Edgington, P. R., McCabe, P., Pidcock, E., Shields, G. P., Taylor, R., Towler, M. & van de Streek, J. (2006). *J. Appl. Cryst.* **39**, 453–457.

[bb10] Markin, A. V., Letyanina, I. A., Ruchenin, V. A., Smirnova, N. N., Gushchin, A. V. & Shashkin, D. V. (2011). *J. Chem. Eng. Data*, **56**, 3657–3662.

[bb11] Sheldrick, G. M. (2008). *Acta Cryst.* A**64**, 112–122. 10.1107/S0108767307043930 18156677

[bb12] Westrip, S. P. (2010). *J. Appl. Cryst.* **43**, 920–925.

